# Research on the decision-making of work safety investment in industrial park enterprises: evidence from behavioral experiments

**DOI:** 10.3389/fpubh.2024.1295536

**Published:** 2024-02-07

**Authors:** SuXia Liu, Shuyue Bao, Daojian Yang, Jingjing Zhang

**Affiliations:** School of Management, Jiangsu University, Zhenjiang, Jiangsu, China

**Keywords:** industrial park enterprises, work safety investment, behavioral experiments, altruistic preference, risk preference

## Abstract

Due to the clustering of risk factors, industrial park safety accidents can easily trigger a domino effect. Work safety investment is the foundation of enterprise work safety in industrial parks. Therefore, increasing the work safety investment of enterprises in industrial parks is the key condition to prevent accidents. However, due to the typical negative externalities of industrial park work safety accidents, the decision-making process of work safety in park enterprises is influenced by other enterprises within the park, including imitation behaviors. This makes the decision-making of work safety in park enterprises very specific. In order to clarify the influencing factors and effects of work safety investment in industrial park enterprises, this study uses a behavioral experiment method and conducts decision-making experiments using the experimental platform O-Tree. The study recruits 76 participants who play the role of decision-makers in park enterprises. This study uses a lottery price experiment and a dictator experiment to measure the risk preference and altruism preference of the participants, respectively. The study introduces the real background of work safety investment in industrial park enterprises and collects data on work safety investment by the participants in different experimental scenarios. The research results show that the safety attitudes of decision-makers, altruism preference, accident experience, government work safety supervision, park management measures, and safety benefits positively influence work safety investment in park enterprises. The risk preference of decision-makers and the resource capability of work safety negatively influence work safety investment in park enterprises. Work safety investment in park enterprises is influenced by the work safety investment of other enterprises within the park.

## 1 Introduction

Industrial parks are a common feature of industrial development and serve as highly concentrated hubs for resources (1, 2). With well-developed infrastructure and low investment costs, industrial parks can provide competitive advantages for enterprises and contribute to the economic development of the regions they are in (3, 4). As industrial parks serve as gathering places for numerous small and medium-sized enterprises, the accumulation of accident risks in such parks exceeds the risks associated with individual operations (5, 6). The establishment of a safety management system within a cluster is associated with the external domino effect (7, 8). In the event of an accident within a single enterprise in the industrial park, it has the potential to initiate a domino effect, thereby increasing the likelihood of subsequent chain accidents (9). Analyzing the risk of the domino effect in uncertain circumstances can aid decision makers in making informed safety investment decisions and identifying the most effective decision strategies in worst-case scenarios. This helps minimize the impact of domino accidents on the enterprises operating in the park (10, 11). Therefore, industrial parks should place greater emphasis on work safety, and park enterprises need to strengthen their work safety investment.

Increasing work safety investments in park enterprises can reduce the probability of work safety accidents and improving work safety investment in industrial park enterprises is the primary condition for preventing accidents in industrial parks (12). However, the decision-making process regarding work safety investment in industrial park enterprises is highly complex. In industrial park settings, many enterprises are small to medium-sized, often facing financial constraints. Therefore, when making decisions regarding work safety investment, these enterprises must consider not only government safety regulations but also the cost–benefit considerations of safety. Additionally, as enterprises operating within an industrial park, they are likely to consider the practices of other companies when making work safety investment decisions. Furthermore, they may also be influenced by the negative externalities associated with accidents.

Additionally, as enterprises within an industrial park, they not only look to other enterprises for reference but are also influenced by the negative externalities of accidents. Therefore, decision-making regarding work safety investments in industrial park enterprises is highly complex.

Previous studies on the influence of work safety investment decision-making in enterprises mainly focused on three aspects: first, government work safety regulatory measures, exploring the positive and negative effects of work safety regulation on promoting proactive work safety investment by enterprises and the burden it imposes on enterprises (13, 14); second, the characteristics of enterprises themselves, such as the safety attitude of decision-makers and the capability of work safety resources (15, 16); third, cost–benefit analysis, for example, Yue (17) introduced the Douglas function to construct a safety investment-safety economic benefit model.

Research on work safety investment decision-making in enterprises has predominantly examined government regulatory measures, corporate attributes, and cost–benefit analysis. At the government level, supervision of work safety by the government influences the safety-sensitive decisions made by enterprises, thereby impacting the likelihood of workplace accidents (18). From both positive and negative perspectives, government safety supervision measures encourage proactive investment in work safety by enterprises, while simultaneously placing additional operational burdens on them (13, 14). Corporate attributes encompass the influence of decision-makers’ attitudes toward safety and the actual capacity of work safety resources within the company (15, 16). Cost–benefit analysis, for instance, as demonstrated by Yue (17), incorporates the Douglas function to construct a safety investment-safety economic benefit model. The analysis found that due to resource limitations, when making work safety investment decisions, enterprises seek a balance between work safety investment and production in order to maximize economic benefits (19, 20). Existing research mainly focuses on analyzing work safety investment decision-making in enterprises, and no research on safety investment decision-making in industrial park enterprises has been found. Based on the characteristic that decision-making power in industrial park enterprises is in the hands of the business owners, this study uses behavioral experiments to study the factors and effects of work safety investment decision-making in park enterprises. Behavioral experiments can measure individual preferences by observing individual behavior (21), create laboratory environments like reality using simulation methods, and obtain experimental data by changing experimental parameters. In this study, the experimental participants take the role of decision-makers in park enterprises and make work safety investment decisions in evolving experimental scenarios. By combining role-playing and parameter settings that are in line with reality, more reliable data can be obtained.

The research object of this study is the decision-makers of industrial parks. The objective is to reduce the frequency of safety accidents in industrial parks and identify the underlying causes and mechanisms affecting work safety investment decision-making. To achieve this purpose, this study constructs realistic scenarios faced by industrial park decision-makers through behavioral experiments and analyzes the factors influencing safety investment decision-making in industrial parks. By analyzing the influencing factors of work safety investment decision-making in industrial parks, effective strategies are provided to improve work safety investment, enhance the work safety status of enterprises, and reduce the occurrence of safety accidents and casualties. The specific structure of this paper is as follows: The second part is literature review and research hypotheses; the third part is experimental design and relevant parameters setting; the fourth part is analysis of experimental results; the fifth part is the discussion and conclusion.

## 2 Literature review and research hypotheses

The work safety investment decision-making of park enterprises is a type of decision-making behavior, where park enterprises decide on the allocation of various resources for work safety. Considering that decision-making power in park enterprises lies in the hands of the business owners, work safety investment decision-making in park enterprises is an individual decision-making behavior of the owners. According to behavioral decision theory, individuals consider intrinsic motivations and external factors when making decisions. Therefore, in addition to considering the cost–benefit of work safety investment from an economic perspective (22), park enterprises’ work safety investment decision-making is influenced by the owners’ intrinsic psychological tendencies, safety attitudes (23), experiences (24), altruistic preferences (25), risk preferences (26), as well as external stakeholders such as government safety supervision departments (14), park management authorities (27), and other enterprises within the park.

### 2.1 Internal influencing factors of work safety investment decision of industrial parks enterprise

#### 2.1.1 The influence of safety costs and benefits

Safety cost refers to the total manpower, material, and financial resources invested in achieving safety. Safety benefits primarily refer to the promotion of economic production value through the maintenance and protection of productivity, reducing or avoiding injuries and losses. Safety benefits have characteristics such as potentiality, indirectness, and lag. When the level of safety investment is constant, enterprises focus on the benefits that the investment brings to the enterprise when making work safety investment decisions (28). The potential and lag characteristics of safety benefits make it difficult for enterprises to perceive and recognize the benefits that safety costs bring to the enterprise (3). When enterprises realize the safety benefits brought by safety costs (29), they will increase work safety investment. Therefore, the following hypotheses are proposed.

*H*1: Decision-makers who perceive safety benefits are more willing to increase work safety investment.

#### 2.1.2 The influence of work safety resource capability

Work safety resource capability refers to the human, financial, facilities, technology, and methodology that an enterprise possesses for implementing work safety. Some parks face issues such as lack of planning, low entry barriers for enterprises, and weak safety foundations. Huang et al. (15) research indicates that safety resource conditions are an important factor influencing enterprise’s work safety decisions. The work safety resource capability of park enterprises impacts their safety conditions and has an influence on the risk of work safety accidents. Park enterprises with strong work safety resource capability have the necessary equipment and facilities for work safety, as well as sufficient financial investment. Therefore, they have a higher level of work safety, and in order to maintain their safety conditions, they increase their work safety investment. Park enterprises with weak work safety resource capability do not have sufficient funds to invest in work safety, resulting in a lower level of work safety investment. Based on this, the following hypothesis is proposed.

*H*2: Industrial Park enterprises with different levels of work safety resource capability have significant differences in work safety investment. Industrial Park enterprises with strong work safety resource capability have higher work safety investment, while enterprises with weak work safety resource capability have lower work safety investment.

#### 2.1.3 The influence of accident experience and safety attitudes

Psychological research suggests that individual experiences are closely related to their behavior and can have long-term effects on their future behavior (30). As significant experiences in life, individual experiences have a significant impact on their thinking patterns and play an important role in decision-making (31). Decision-makers in enterprises with accident experiences are aware of the concept that work safety investment is lower than the cost of accidents, and they increase work safety investment to avoid accidents. On the other hand, decision-makers without accident experiences are more likely to overlook the necessity of work safety investment, resulting in a lack of motivation for work safety investment.

Safety attitude refers to the inherent response tendencies individuals have toward various safety issues under the guidance of safety values. It represents their thoughts and confidence in safety goals and tasks. Safety attitude is an important factor in work safety decision-making (16). Managers have latent and unobservable mental states about safety (32). Liu et al. (33) argues that the safety attitude of managers in enterprises affects the establishment of work safety goals, and managers with positive safety attitudes are more willing to support work safety behaviors in the enterprise. Therefore, the following hypotheses are proposed.

*H*3: The accident experience of decision-makers positively affects work safety investment decision-making in industrial park enterprises.

*H*4: The safety attitudes of decision-makers positively affect work safety investment decision-making in industrial park enterprises.

#### 2.1.4 The influence of risk preferences

Risk preferences reflect an individual’s attitude toward risk and are an important indicator for predicting personal behavior or choices (34, 35). They can be divided into two types: risk-seeking and risk-averse. When making decisions, individuals are influenced by the attitude toward risk presented at that time (36). Both risk-seeking and risk-averse preferences have important effects on decision-making behavior. Risk-seeking decision-makers tend to take more risky behavior when making decisions (37). Therefore, when faced with the risk of work safety accidents, industrial park owners with different risk preferences will make different work safety investment decisions. Risk-averse decision-makers will choose to increase work safety investment to avoid the serious losses caused by accidents. Risk-seeking decision-makers will focus on production revenue and choose to reduce enterprise work safety investment. Therefore, this study proposes the following hypotheses:

*H*5: Industrial Park enterprises decision-makers with risk-seeking preferences will reduce work safety investment.

*H*6: Industrial Park enterprises decision-makers with risk-averse preferences will increase work safety investment.

#### 2.1.5 The influence of altruistic preferences

In traditional economics, it is assumed that individuals are rational. However, the rise of behavioral economics has led economists to explore the role of individuals’ social attributes in decision-making (38). Altruistic preferences reflect individuals’ social nature. Altruistic preferences refer to individuals unilaterally engaging in beneficial behavior toward others (39). According to the theory of altruistic preferences, individual decision-making deviates from the rational economic agent assumption of traditional pursuit of self-interest maximization. When making decisions, individuals not only consider their own benefits but also consider the benefits of others. Due to the agglomeration of park enterprises, when accidents occur in one enterprise, it not only causes losses to the affected enterprise but may also trigger a “domino effect” of accidents that affects other non-accident enterprises within the park (40). In severe cases, it may lead to the escalation of accidents, causing major disasters (9). For entrepreneurs with altruistic preferences, when making decisions regarding work safety investment, they consider not only their own enterprise’s work safety and accident risks but also the impact of accident risks on neighboring enterprises and even the entire park. Considering the negative external spillover effects of accidents in the park, this study proposes the following hypothesis:

*H*7: Under negative externalities, industrial park decision-makers with altruistic preferences will increase work safety investment.

### 2.2 External influencing factors of work safety investment decision of industrial parks enterprise

#### 2.2.1 The influence of government work safety regulation

The impact of government work safety regulation includes work safety investment subsidies, work safety inspections, and penalties for violations. Enterprise work safety is closely linked to government regulation, and government regulatory measures influence the decision-making process of businesses regarding work safety (18). Positive incentive measures, such as work safety investment subsidies, implemented by the government can encourage businesses to increase their investment in work safety (41, 42). By intensifying work safety inspections, the government can deter businesses from adopting a complacent attitude toward work safety and encourage them to allocate more resources to ensure safety. Imposing penalties on enterprises for work safety violations aims to discourage them from taking risks and engaging in unsafe practices, ultimately fostering increased investment in work safety. Based on these considerations, this study proposes the following hypotheses:

*H*8: There are significant differences in industrial park enterprise work safety investment under different government incentives and sanctions.

*H*8a: Government special subsidies for work safety investment have a positive impact on enterprise work safety investment in the industrial park.

*H*8b: Government work safety inspection has a positive impact on enterprise work safety investment in the industrial park.

*H*8c: Government punishment for work safety violations has a positive impact on enterprise work safety investment in the industrial park.

#### 2.2.2 The influence of work safety management in industrial park

Once a work safety accident occurs in an industrial park, it not only impacts the reputation of the park but also affects the overall environmental safety within the park (43). In order to maintain a safe ecological environment within the park, the management authorities implement work safety management measures for the enterprises operating in the park. The management authorities utilize a combination of support and supervision to promote and actively involve these enterprises in the construction of a safe environment. The support aspect includes organizing work safety training programs and providing subsidies to encourage enterprises to introduce advanced work safety equipment (44). On the other hand, supervision involves conducting regular work safety inspections to investigate potential safety hazards and supervise enterprises to promptly rectify any issues. The support and supervision approach adopted by the management authorities motivates enterprises in the industrial park to place a significant emphasis on work safety and increase their investment in this area. Therefore, based on these considerations, this study posits the following hypothesis:

*H*9: Industrial park work safety management has a positive impact on the work safety investment of park enterprises.

#### 2.2.3 The influence of other enterprises in the industrial park

The reference dependence of decision-making individuals can influence their decision-making behavior (45). Individuals tend to unconsciously establish a reference point before making decisions and make decisions based on this reference point (46). In the field of behavioral decision-making, reference points play a significant role in explaining the status quo bias (46). Individuals establish their reference points by comparing themselves to others. In an industrial park (47), where multiple enterprises operate in the same industry, the work safety investment decisions of these enterprises are likely to be influenced by the work safety investments of other enterprises within the park, which serve as their reference points. Therefore, based on these considerations, this study proposes the following hypothesis:

*H*10: The work safety investment decisions of industrial park enterprises are influenced by the work safety investments of other enterprises within the park.

## 3 Experimental design and relevant parameters setting

### 3.1 The theoretical framework for measuring individual risk preference and altruistic preference

In this study, participants’ risk preferences were measured based on Holt’s lottery pricing experiment (48). The experiment choices are shown in Table 1, where participants made choices between 10 pairs of lotteries. The lotteries were categorized as A and B. Lottery A had a potential payoff of either 2 or 1.6, while lottery B had a potential payoff of either 3.85 or 0.1. Lottery A can be considered as the safe option, while lottery B is the risky option. Therefore, by observing the participants’ preference shift from option A to option B, it is possible to evaluate their level of risk aversion.

**Table 1:** Lottery pricing experiment options.

	Option A	Option B
1	1/10, 2; 9/10, 1.6	1/10, 3.85; 9/10, 0.1
2	2/10, 2; 8/10, 1.6	2/10, 3.85; 8/10, 0.1
3	3/10, 2; 7/10, 1.6	3/10, 3.85; 7/10, 0.1
4	4/10, 2; 6/10, 1.6	4/10, 3.85; 6/10, 0.1
5	5/10, 2; 5/10, 1.6	5/10, 3.85; 5/10, 0.1
6	6/10, 2; 4/10, 1.6	6/10, 3.85; 4/10, 0.1
7	7/10, 2; 3/10, 1.6	7/10, 3.85; 3/10, 0.1
8	8/10, 2; 2/10, 1.6	8/10, 3.85; 2/10, 0.1
9	9/10, 2; 1/10, 1.6	9/10, 3.85; 1/10, 0.1
10	10/10, 2; 0/10, 1.6	10/10, 3.85; 0/10, 0.1

In this study, participants’ altruistic preferences were measured through a dictator experiment (49). In this experiment, participants were assigned either the role of a dictator or a receiver. Under complete anonymity, the dictator proposed a distribution plan for a sum of money, and the receiver could only choose to accept it. In this study’s experiment, the dictator allocated 10 units of money. If the dictator allocated any amount higher than zero to the receiver, it indicated that the dictator had altruistic preferences.

### 3.2 The organization of the experiment, the selection of subjects, and their reward incentives

The experiment was conducted in two batches. The first batch consisted of highly cognitive and academically capable undergraduate students from universities. The second batch consisted of experienced corporate managers who were familiar with enterprise management decision-making. All participants volunteered to participate in the experiment and had no prior involvement in related decision-making experiments. Before the experiment began, the experimenter provided detailed instructions on the experimental procedure and explained how the final earnings would be calculated.

The experiment used monetary incentives to motivate participants to make real decisions. Prior to the experiment, participants were informed that their decisions in the experiment would determine their earnings, which would ultimately be converted into cash rewards. After completing all rounds of the experiment, the computer automatically calculated the experimental earnings for each participant. The experimental earnings consisted of two components: a participation fee for taking part in the experiment and performance-based earnings for the participant’s performance in the experiment.

### 3.3 Experimental process and related parameter settings

Before the formal start of the experiment, participants were first provided with the experiment instructions to read and familiarize themselves with the experimental procedure. After gaining understanding of the experiment flow, the experiment officially began. After collecting basic information from the participants, they will undergo measurements of safety attitudes, individual preferences, and the dictator game. Additionally, they will participate in an experiment involving decision-making regarding investment in work safety in industrial park enterprises, which includes 12 different experimental scenarios.

#### 3.3.1 Read the experiment instructions

The experimenter distributes the experiment instructions and provides an explanation before the start of the experiment to ensure that participants are familiar with the experimental procedure and to ensure the smooth running of the experiment. The experimenter emphasizes the following points: Firstly, anonymity. The participants will be randomly assigned to groups by the computer, and they will not know the identities of other members within their group. Secondly, independence. Participants are instructed to complete all steps independently and are prohibited from communicating with other participants. Thirdly, privacy. The experimenter will be responsible for calculating the participants’ earnings, and only the participants themselves will know their own earnings.

#### 3.3.2 Experimental measurement of safety attitudes and individual preferences

The first part consists of a survey on personal information, including gender, age, and mobile phone number for the purpose of final payment settlement. The second part involves the measurement of individual safety attitudes. In this part, participants’ safety attitudes are tested using five questionnaire items. The third part measures individual preferences, specifically the measurement of risk preferences and altruistic preferences. Risk preferences are measured through a lottery price experiment, while altruistic preferences are measured through a dictator experiment.

#### 3.3.3 Experimental study on work safety investment decisions of enterprises in industrial parks under different experimental scenarios

This study utilizes the O-Tree experimental platform to design a work safety investment decision experiment for different scenarios in industrial parks. Each round of the experiment maintains the same initial resources, and the remaining resources and earnings from the previous round are not considered in the next round of the experiment. This research aims to simulate realistic scenarios of work safety investment decision-making in industrial parks under different experimental conditions. Participants in the experiment play the role of decision-makers in industrial park enterprises. The purpose of the experiment is to analyze the influence of various factors on work safety investment decision-making in industrial park enterprises under different scenarios. The settings for different experiment scenarios are shown in Table 2.

(1) Control experiment without additional incentives or penalties: E1

**Table 2:** Explanations of different experimental scenarios.

Experiment name	Experimental scenarios
E1	Control experiment without additional rewards or punishments.
E2	Enterprises in the industrial park have different capabilities in work safety resources
E3	Government’s special subsidy for work safety in industrial parks’ enterprises
E4.1	Government’s weak inspection efforts on work safety in industrial parks’ enterprises
E4.2	Government’s strong inspection efforts on work safety in industrial parks’ enterprises
E5	Government’s punishment for production violations in industrial parks’ enterprises
E6	The intervention of the industrial park management in the work safety management of enterprises in the park
E7.1	The group consists of no additional incentives or penalties, and the work safety investment is displayed.
E7.2	
E8	Negative externalities of industrial park enterprise accidents
E9.1	The group consists of no additional incentives or penalties, and the earnings and safety conditions are displayed.
E9.2	

This control experiment is set up as a benchmark for comparison with other experimental groups. In the experiment, participants are randomly assigned into groups of 4 individuals and engage in a one-round decision-making process regarding work safety investment in industrial park enterprises. Each participant starts with an initial capital of 4,000, and after deciding on the amount of work safety investment, the remaining capital will be treated as an investment and generate a return at a rate of 20%. The work safety conditions of the enterprise are directly proportional to the amount of work safety investment. There are no penalties in this round of the experiment, and all members of the control experiment have the same baseline conditions. At the end of the experiment, participants are not informed about the group’s investment amount or their own individual earnings for the round.

(2) Experiment with different benchmarks: E2

In this round of the experiment, the enterprises represented by all participants are randomly divided into four levels of work safety levels, namely A, B, C, and D, from high to low. Participants will make work safety investment decisions under the conditions of no additional incentives or penalties and different safety levels. At the end of the experiment, participants are not informed about the group’s investment amount or their own individual earnings for the round.

(3) Experiments with incentives and penalties: E3, E4.1, E4.2, E5, E6, E8

The purpose of setting up these experimental groups is mainly to consider the influence of government safety regulation and park safety management on work safety investment decisions in industrial park enterprises. The experiment consists of 5 rounds, including the following elements: government subsidies for work safety investment, government safety inspections, government fines for accident-prone enterprises, government orders for production suspension and rectification of accident-prone parks, and park management’s requirement for enterprises to participate in safety training. In these 5 rounds, participants will make work safety investment decisions based on different incentives, penalties, and management requirements. All participants in the 5 rounds of the experiment have the same baseline conditions. At the end of the experiment, participants are not informed about the group’s investment amount or their own individual earnings for each round.

(4) Experiments with the same benchmark and result display: E7.1, E7.2, E9.1, E9.

In all four experiments, all participants have the same baseline conditions with no additional penalties, but there is a certain level of result display after the experiment. In experiment E7.1, participants’ work safety investment within their respective groups will be shown, followed by experiment E7.2 with the same parameter settings. After the decision-making phase of experiment E9.1, the participants’ financial gains and the safety status of the company (whether any safety accidents occurred) were presented. Subsequently, the participants were exposed to experiment E9.2, which had the same scenario settings as experiment E9.1, but this time they were informed about the work safety investment results of experiment E9.1.

## 4 Analysis of experimental results

This experiment consists of two sessions. The first session is an offline experiment conducted by recruiting undergraduate students. The experiment took place on March 10th from 10:00 to 11:00 in Room 1,205 of Sanjiang Building, Jiangsu University. A total of 48 undergraduate students were recruited, forming 12 groups. After excluding one group with 4 missing samples, a total of 44 valid data were collected. In order to enhance sample diversity and increase data reliability, the experiment also recruited 28 enterprise managers with certain social work experience through online recruitment. They were divided into 7 groups and the experiment started on April 10th at 19:00.

The two sessions of the experiment had different participant categories, timings, and locations. Referring to Siegel (50), non-parametric test methods were used, and the Wilcoxon Mann–Whitney test was employed to analyze whether there were significant differences in work safety investment between the two sessions. The results of the test for differences between the two sessions are presented in Table 3.

**Table 3:** Two experiments Wilcoxon Mann–Whitney nonparametric test.

Experimental name	Variable value	Sample size	Median	Standard deviation	Statistic	*p*	Median value difference	Cohen’s d
E1	Student	44	120	51.355	646	0.727	1.5	0.007
	Business manager	28	118.5	21.929				
E2	Student	44	150	63.337	627.5	0.894	0	0.08
	Business manager	28	150	50.472				
E3	Student	44	140	51.276	759	0.097	15	0.188
	Business manager	28	125	20.037				
E 4.1	Student	44	150	46.436	759	0.096	30	0.371
	Business manager	28	120	27.585				
E4.2	Student	44	160	41.755	770.5	0.073	20	0.316
	Business manager	28	140	23.992				
E5	Student	44	187.5	50.201	622.5	0.938	27.5	0.196
	Business manager	28	160	29.073				
E6	Student	44	145	42.477	703	0.312	7.5	0.07
	Business manager	28	137.5	24.471				
E7.1	Student	44	150	55.431	668.5	0.542	20	0.066
	Business manager	28	130	29.252				
E7.2	Student	44	160	25.452	697.5	0.342	7.5	0.204
	Business manager	28	152.5	25.509				
E8	Student	44	170	44.15	760.5	0.093	20	0.306
	Business manager	28	150	37.552				
E9.1	Student	44	170	39.423	738.5	0.154	27.5	0.331
	Business manager	28	142.5	36.009				
E9.2	Student	44	195	32.555	751.5	0.101	15	0.497
	Business manager	28	180	55.965				

According to Table 3, the non-parametric test using Wilcoxon Mann–Whitney shows that for all 12 sessions (E1-E9.2) of the experiment, the *p*-values are greater than 0.05. Therefore, the null hypothesis is accepted, indicating that there is no significant difference between the sample data from the two sessions. As a result, this study can combine the data from both sessions for the analysis of work safety investment decision-making in enterprises.

### 4.1 Analysis of the overall data population

**Figure 1:**
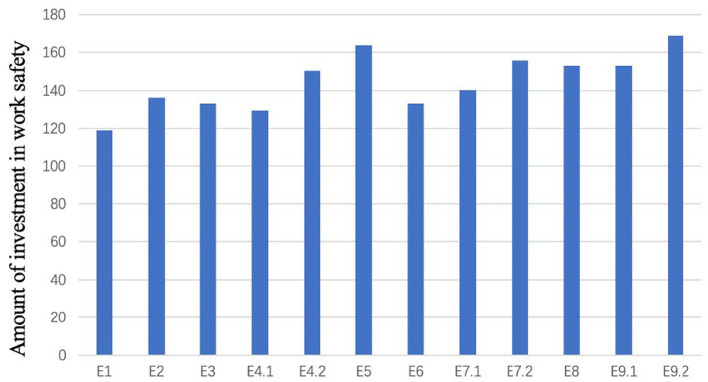
The mean work safety investment under different experimental scenarios.

Seventy-two participants were involved in the experiment on work safety investment decision-making. In the 12 sessions of the experiment, participants were given the option to choose work safety investment amounts ranging from 0 to 200. The participants were randomly divided into groups of four. This study aimed to provide descriptive statistics of work safety investments made by participants in different experimental scenarios, as well as to conduct differential tests to observe their decision-making behavior regarding work safety investments across various scenarios. The mean work safety investment amounts in different experimental scenarios and the results of the Friedman test are presented in Figure 1 and Table 4, respectively.

**Table 4:** Friedman test of different scenarios.

Experimental name	Sample size	25-digit	median	75-digit	The statistic χ^2^ value	*p*
E1	72	100.000	119.500	140.000	208.397	0.000
E2	72	97.500	150.000	180.000		
E3	72	115.000	130.000	150.000		
E4.1	72	100.000	124.500	156.250		
E4.2	72	125.000	150.000	180.000		
E5	72	150.000	180.000	200.000		
E6	72	120.000	140.000	160.000		
E7.1	72	100.000	147.500	180.000		
E7.2	72	143.750	160.000	170.000		
E8	72	127.500	160.000	182.500		
E9.1	72	122.250	160.000	180.000		
E9.2	72	150.000	185.000	200.000		

According to Figure 1, it can be observed that the work safety investments made by participants in different experimental scenarios exhibit some fluctuation. Additionally, the mean work safety investment amounts exceed 100 in all experimental scenarios. E1, serving as the control experiment without any additional rewards or penalties, has the lowest average work safety investment value among all work safety investment decision-making experiments. On the other hand, E9.2 has the highest mean work safety investment amount among all experimental scenarios.

From Table 4, it can be observed that there are significant differences in work safety investments made by participants in different experimental scenarios. Table 4 presents the non-parametric tests of the work safety investments made by participants over the 12 sessions. Through the Friedman test, it was investigated whether there were differences in the work safety investment data across the 12 sessions. The test results revealed that the value of p is less than 0.05, indicating the rejection of the null hypothesis. This suggests that there are significant differences in work safety investments made by participants over the 12 sessions.

### 4.2 Analysis of participants’ safety attitudes

Participants’ safety attitudes and individual preferences influence work safety investment decisions. In this study, before participants engaged in the work safety investment decision experiment, their safety attitudes were measured through a questionnaire, their risk preferences were measured through a lottery pricing experiment, and their altruistic preferences were measured through a dictator game experiment.

To validate the influence of participants’ safety attitudes on work safety investments in the industrial park, it is necessary to assess the reliability and validity of the questionnaire data. Reliability is assessed using Cronbach’s alpha coefficient, with values ranging from 0.7 to 0.98 indicating high reliability. Validity assessment involved the Kaiser-Meyer-Olkin (KMO) measure and the Bartlett’s Test of Sphericity. The results of the reliability and validity tests for the safety attitudes questionnaire are presented in Table 5.

**Table 5:** Reliability and validity test of safety attitude questionnaire.

Cronbach’s α	KMO	Bartlett’s test of sphericity			Approximate chi-square	*df*	*p*
0.831	0.795	139.298	10	0.000

Table 5 indicates that the questionnaire has high reliability, with a Cronbach’s alpha coefficient within the acceptable range. The KMO value of 0.795 and the significant value of p (< 0.01) from the Bartlett’s Test of Sphericity demonstrate good structural validity of the questionnaire.

This study examines the impact of safety attitudes of decision makers on work safety investment in industrial park enterprises. E1 was used as a control experiment without additional incentives, and this study conducted regression analysis on the safety attitude and work safety investment of the participants in E1 experiment. The regression results are presented in Table 6.

**Table 6:** Regression results of safety attitudes.

	Nonstandardized coefficient		Standardization coefficient	t	*p*	F
	B	Standard error	Beta			
constant	−18.214	31.972	–	−0.57	0.571	F = 18.778 (*p* = 0.000)
safety attitude	30.812	7.11	0.46	4.333	0.000	

From Table 6, it can be observed that the regression model passes the F-test (*F* = 18.778, *p* < 0.05), indicating that safety attitude has a significant impact on work safety investment. The significance analysis of safety attitude on work safety investment reveals a value of p less than 0.05 and a positive standardized coefficient, indicating a positive influence of safety attitude on work safety investment. The higher the safety attitude, the higher the level of work safety investment. Therefore, hypothesis H4 is supported by the results of the analysis.

### 4.3 Analysis of participants’ risk preferences

The present study used participants’ decision-making jump points in a lottery price experiment as an indicator of their risk preferences. This paper make a regression analysis on the risk preference of the research object and its E1 work safety investment. The results of the regression analysis are presented in Table 7.

**Table 7:** Linear regression results of risk preference.

	Nonstandardized coefficient		Standardization coefficient	t	*p*	F
	B	Standard error	Beta			
constant	188.379	12.16	–	15.492	0.000	F = 36.648 (P = 0.000)
risk preference	−20.065	3.315	−0.586	−6.054	0.000	

According to Table 7, the regression model shows a significant impact of risk preferences on work safety investment based on the F-test (*F* = 36.648, *p* < 0.05). The significance analysis of risk preferences on work safety investment indicates a value of p less than 0.05 and a negative standardized coefficient. This suggests that risk preferences do influence work safety investment. Specifically, higher risk preferences are associated with lower levels of work safety investment, while lower risk preferences are associated with higher levels of work safety investment. Hypotheses H5 and H6 have passed the test, indicating that risk preference-oriented decision-makers reduce work safety investment, while risk-averse decision-makers increase work safety investment.

### 4.4 Analysis of participants’ altruistic preferences

This study measured participants’ altruistic preferences through a dictator experiment, where the allocator chose to allocate a positive amount to the recipient, indicating the presence of altruistic preferences. The experiment E8 represents a scenario in which accidents in the industrial park generate negative externalities, causing losses to non-accident enterprises. This study analyzed the impact of altruistic preferences on work safety investment in the E8 scenario by constructing a regression model. The model was constructed as inv. = m + nx, where inv. represents work safety investment, and x is a dummy variable representing participants’ altruistic preferences. When x is 0, it indicates that participants do not have altruistic preferences, and when x is 1, it indicates that participants have altruistic preferences. The regression results are presented in Table 8.

**Table 8:** Linear regression results of altruistic preferences.

	Non standardized coefficient		Standardization coefficient	t	*p*	F
	B	Standard error	Beta			
Constant	132.5	7.754	–	17.088	0.000	*F* = 17.582 (P = 0.000)
altruism preference	41.591	9.919	0.584	4.193	0.000	

Through regression analysis of the model, the results obtained from Table 8 indicate that the model passed the F-test. The significance analysis of altruistic preferences on work safety investment shows a value of p less than 0.05 and a standardized coefficient greater than 0. This indicates that altruistic preferences have a positive impact on work safety investment. Hypothesis H7 has passed the test, suggesting that participants with altruistic preferences will increase work safety investment in the context of negative externalities.

### 4.5 Difference analysis of work safety investment in industrial park enterprises under different experiment scenarios

In this study, a paired Wilcoxon signed-rank test was conducted to examine the work safety investment of participants under different experimental scenarios. E3 and E6 were used to test the impact of safety costs on work safety investment. E9.1 and E9.2 were used to test the impact of safety benefits on work safety investment. E9.1-accident and E9.2-accident were used to examine the influence of participants’ accident experiences on work safety investment. E1 was paired with E3, E1 with E4.1, E1 with E4.2, E1 with E5, and E1 with E6 to test the effects of government work safety subsidies, weak government work safety supervision, strong government work safety inspection, government penalties for work safety violations, and park management work safety management on work safety investment, respectively. E7.1 and E7.2 were used to examine the effects of work safety investment decisions by other enterprises within the industrial park on work safety investment. The results of the paired differences in work safety investment for different experimental scenarios are shown in Table 9.

**Table 9:** Wilcoxon symbolic rank test of paired samples.

Pairing variable	Median ± standard deviation			*z*	*df*	*p*	Cohen’s d
	Pair 1	Pair 2	Pairing difference				
E9.1 & E9.2	167.5 ± 37.897	185 ± 34.675	−20 ± 32.728	4.321	53	0.000	0.512
E9.1-accident & E9.2-accident	157.5 ± 37.927	195 ± 29.898	−20 ± 29.776	5.426	53	0.000	0.781
E1 & E3	119.5 ± 42.192	130 ± 41.953	−10 ± 37.576	4.067	71	0.000	0.337
E1 & E4.1	119.5 ± 42.192	124.5 ± 40.606	−10 ± 47.443	2.416	71	0.016	0.255
E1 & E4.2	119.5 ± 42.192	150 ± 36.138	−30 ± 46.556	5.495	71	0.000	0.796
E1 & E5	119.5 ± 42.192	180 ± 43.186	−40 ± 46.802	6.126	71	0.000	1.05
E1 & E6	119.5 ± 42.192	140 ± 36.36	−20 ± 35.598	5.253	71	0.000	0.436
E7.1 & E7.2	147.5 ± 46.783	160 ± 25.422	−10 ± 39.556	2.974	71	0.003	0.411
E9.1 & E9.2	167.5 ± 37.897	185 ± 34.675	−20 ± 32.728	4.321	53	0.000	0.512

#### 4.5.1 Safety benefits

Safety benefits are characterized by their indirectness and potentiality. They are manifested through the reduction of personnel injuries and property losses caused by accidents. Therefore, enterprises often have difficulty recognizing the value of safety benefits. However, once enterprises realize the value that safety benefits bring to the organization, it can have a significant impact on their work safety investment. Experiment E9.1 and E9.2 represent the pre- and post-cognition of safety benefits by participants. The Mann–Whitney U test was conducted to compare the work safety investment of participants in E9.1 and E9.2. The data analysis results indicate that *p* < 0.05, suggesting a significant difference in work safety investment between E9.1 and E9.2.

#### 4.5.2 Work safety resource capability

The work safety resource capability of enterprises in the industrial park affects their work safety investment. In Experiment E2, participants’ work safety resource capability was categorized from high to low as A, B, C, D. This study conducted a Friedman test on participants’ work safety investment in Experiment E2. The results of the test are shown in Table 10.

**Table 10:** Friedman test for work safety resource capability.

Variable name	Sample size	size median	standard deviation	statistic	*p*	Cohen’s f
E2-A	18	85	52.034	23.086	0.000	0.721
E2-B	18	160	41.815			
E2-C	18	155	47.711			
E2-D	18	190	51.229			

From Table 10, it can be observed that p < 0.05, indicating that there are significant differences in work safety investment among participants with different work safety resource capability.

#### 4.5.3 Accident experience

In this study, E9.1-accident represents work safety accidents that occurred after participants made work safety investments, while E9.2-accident represents work safety accidents that occurred before participants made subsequent round of work safety investments. By conducting a Mann–Whitney U test on the two rounds of experiments, we found that the results were statistically significant with a value of p of less than 0.05. This indicates that there are differences in work safety investment decisions between participants with and without accident experience. Overall, accident experience can affect participants’ decisions regarding work safety investments.

#### 4.5.4 Government reward and punishment measures

Participants showed differences in work safety investment under different government reward and punishment measures. Experiment E3, E4.1, E4.2, and E5 represent the government’s special subsidies for work safety, weak work safety inspection efforts, strong work safety inspection efforts, and penalties for work safety violations, respectively. This study conducted a Friedman test on participants’ work safety investment under different government reward and punishment measures, and the results of the test can be seen in Table 11.

**Table 11:** Friedman test for different government incentives and penalties.

Variable name	Sample size	Size median	Standard deviation	Statistic	*p*	Cohen’s f
E3	72	130	41.953	86.422	0.000	0.342
E4.1	72	124.5	40.606			
E4.2	72	150	36.138			
E5	72	180	43.186			

The results from Table 11 demonstrate significant differences in work safety investments among participants when government reward and punishment measures, such as work safety subsidies, intensified work safety inspections, and penalties for work safety violations, are implemented. Hypothesis H9 has been confirmed. To further investigate these differences, work safety investments of participants in the experimental control E1 (without rewards and punishments) were compared to those in E3, E4.1, E4.2, and E5. The results, presented in Table 9, reveal that all value of *p*s are below 0.05. This indicates that government work safety subsidies, intensified work safety inspections, and penalties for work safety violations have a significant impact on work safety investments.

#### 4.5.5 Industrial park management party work safety supervision

The work safety management by the park management affects the work safety investment of the park enterprises. The E6 represents the work safety management by the park management. According to Table 9, the work safety investment of E6 was subjected to a Mann–Whitney U test with E1. The test results indicate a value of *p* less than 0.05, suggesting that the work safety investment of the participants is influenced by the park management. This indicates that the work safety management by the park management has an impact on the work safety investment of the park enterprises.

#### 4.5.6 Work safety investment of other enterprises in industrial park

**Figure 2:**
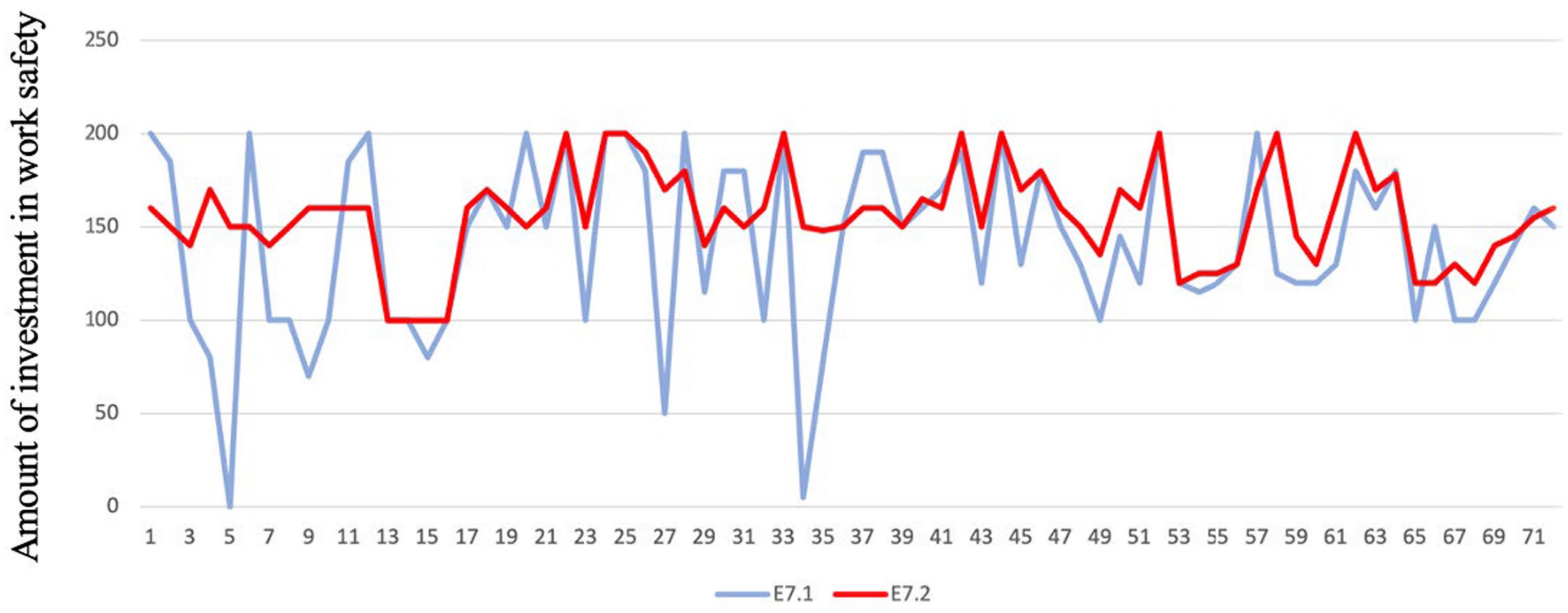
Work safety investment of E7.1 and E7.2.

The work safety investment of enterprises in the park is influenced by the work safety investment of other enterprises in the park. After the work safety investment of the participants in experiment E7.1 is completed, they will be informed of the work safety investment status within their group. Following this, experiment E7.2 will be conducted. The work safety investments of the participants in experiments E7.1 and E7.2 are compared using the Mann–Whitney U test. The results show that *p* < 0.05, indicating that there is a significant difference in the work safety investments of the participants between experiments E7.1 and E7.2. This suggests that the decision-making regarding work safety investment of enterprises in the park is influenced by the work safety investment of other enterprises in the park. Figure 2 compares the work safety investments of the participants in E7.1 and E7.2, showing that the difference in work safety investment among the participants in E7.2 is smaller compared to E7.1. The participants refer to the work safety investments of their group members and reduce the gap between their own work safety investment and that of the group members. Hypothesis H10 is validated through this test.

### 4.6 Regression analysis of influencing factors of work safety investment decision of industrial park enterprises under different experimental scenarios

Based on the analysis above, it can be concluded that safety benefits, accident experience, government rewards and penalties, and safety management by the park management have an impact on work safety investment by enterprises in the park. In this section, a regression equation (Equation 1) is constructed to further analyze the linear relationship between safety benefits, work safety resource capability, accident experience, government rewards and penalties, park safety management, and work safety investment.

(1) $$inv=\alpha_{i}+\beta_{i}x_{i}$$

Inv represents the amount of work safety investment made by individual decision-makers, where i can take values 1, 2, 3, 4, 5, 6, 7 (1 represents safety benefits, 2 represents enterprise work safety resource capability, 3 represents accident experience, 4 represents government special subsidies for work safety, 5 represents government inspection intensity for work safety, 6 represents government penalties for work safety violations, and 7 represents park work safety management). $x_{i}=0,1$ , where $x_{i}$ is a dummy variable indicating whether a specific scenario has occurred. When $x_{i}$ =0, it means that the corresponding scenario has not occurred, and when $x_{i}$ =1, it means that the scenario has occurred. $\beta_{i}$ represents the extent to which $x_{i}$ affects work safety investment. When $\beta_{i}$ > 0, it indicates that the occurrence of the corresponding scenario increases the decision-maker’s investment in work safety, while $\beta_{i}$ < 0 indicates that the occurrence of the corresponding scenario decreases the decision-maker’s investment in work safety.

The linear regression results for safety benefits, enterprise work safety resource capability, accident experience, government rewards and penalties (government special subsidies for work safety, government inspection intensity for work safety, government penalties for work safety violations), and park work safety management on work safety investment are shown in Table 12.

**Table 12:** The linear regression results of work safety investment under different experimental scenarios.

		Non standardized coefficient		Standardized coefficient	F
		B	Standard error	Beta	
Safety benefits	$\alpha_{1}$	152.833	4.871	–	*F* = 5.47 (*p* = 0.021)
	$\beta_{1}$	16.111	6.888	0.193	
Enterprise work safety resource capability	$\alpha_{2}$	197.556	14.717	–	*F* = 22.981 (P = 0.000)
	$\beta_{2}$	−25.761	5.374	−0.497	
Accident experience	$\alpha_{2}$	151.278	4.647	–	*F* = 16.487 (P = 0.000)
	$\beta_{2}$	26.685	6.572	0.367	
Government special subsidies for work safety	$\alpha_{3}$	118.986	4.958	–	*F* = 4.098 (*p* = 0.045)
	$\beta_{3}$	14.194	7.012	0.167	
Government inspection efforts on work safety	$\alpha_{4}$	129.542	4.53	–	*F* = 10.435 (*p* = 0.002)
	$\beta_{4}$	20.694	6.406	0.262	
Government penalties for work safety violations	$\alpha_{5}$	118.986	5.031	–	*F* = 39.702 (P = 0.000)
	$\beta_{5}$	44.833	7.115	0.467	
Industrial park work safety management	$\alpha_{6}$	118.986	4.641	–	*F* = 6.829 (*p* = 0.010)
	$\beta_{6}$	17.153	6.564	0.214	

As can be seen from Table 12. Safety benefits have a significant effect on work safety investment, with a value of p less than 0.05 and a positive standardized coefficient. This indicates that participants, who are aware of the benefits of safety investment, would increase their investment in work safety. Hypothesis H1 passed the test. Enterprises work safety resource capability has a significant effect on work safety investment, with a value of p less than 0.05 and a negative standardized coefficient. This suggests that participants may overlook work safety investment when facing strong work safety resource capability, possibly due to a sense of confidence in their own work safety condition. On the other hand, participants may increase work safety investment when facing weak work safety resource capability, out of fear of future safety accidents. This would enhance enterprise work safety resource capability and reduce the likelihood of future safety incidents. Hypothesis H2 did not pass the test. Accident experience had a significant effect on work safety investment, with a value of p less than 0.05 and a positive standardized coefficient. This indicates that participants with accident experience would increase their investment in work safety. Hypothesis H3 passed the test. Government special subsidies for work safety had a significant effect on work safety investment, with a value of p less than 0.05 and a positive standardized coefficient. This suggests that participants would increase their investment in work safety due to government special subsidies. Hypothesis H8a passed the test. Government inspection intensity for work safety had a significant effect on work safety investment, with a value of p less than 0.05 and a positive standardized coefficient. This indicates that participants would increase their work safety investment with increasing government inspection intensity. Hypothesis H8b passed the test. Government penalties for work safety violations had a significant effect on work safety investment, with a value of p less than 0.05 and a positive standardized coefficient. This suggests that participants would increase their work safety investment due to government penalties. Hypothesis H8c passed the test.

Industrial park management party has a significant impact on work safety investment, *p* < 0.05, the standardization coefficient is positive. This indicates that participants will increase investment in work safety because of work safety management of industrial park. Hypothesis H9 passed the test.

## 5 Conclusion

### 5.1 Research conclusion and management implications

Through the analysis of the differences in safety investment between undergraduate students and experienced managers in the workplace, it was found that there is no significant difference in safety investment decisions between the two groups in different experimental scenarios. This study examines the differential factors influencing safety investment decisions in various experimental situations. The results indicate that government rewards and penalties, safety management within the industrial park, decisions made by other companies in the park, safety benefits, the resources and capabilities of work safety within the enterprise, and the safety attitudes, accident experiences, and individual preferences (including risk preferences and altruistic preferences) of decision-makers significantly influence safety investment decisions.

Furthermore, a regression analysis was conducted to identify the factors that influence safety investment decisions in the park. The study found that decision-makers increase safety investment when they recognize the benefits of safety income to the company. Safety attitudes, altruistic preferences, and accident experiences positively affect safety investment decisions, while risk preferences have a negative impact. Decision-makers with altruistic preferences tend to increase safety investment when facing negative external effects of safety accidents. Additionally, the resources and capabilities of work safety within the enterprise have a negative influence on safety investment.

Therefore, park enterprises should prioritize long-term interests and consider increasing safety investment as a prerequisite for achieving long-term benefits and further development. Decision-makers in park enterprises should adopt a positive attitude and recognize the safety benefits brought by safety investment, actively addressing work safety issues. Considering the spatial relationships among enterprises within the park, accidents can have negative external effects that connect all companies economically. Park enterprises should consciously cooperate with safety inspections, eliminate hidden dangers, actively shoulder work safety responsibilities, and promote a mutually beneficial and altruistic atmosphere for safety investment in collaboration with the park management. Park enterprises should not relax work safety regulation or reduce safety investment based on favorable safety conditions. They should understand that safety investment is crucial for ensuring long-term stability and development.

Regression analysis of the government factors influencing safety investment decisions in the park revealed that government subsidies for safety investment, safety inspections, and penalties for safety violations have a positive impact on safety investment in park enterprises. Therefore, at the government level, work safety regulation measures can effectively promote work safety in enterprises. Government subsidies for work safety can enhance the enthusiasm of enterprises to invest in safety. Safety inspections by the government can prevent enterprises from having a careless attitude toward safety and reducing safety investment. Administrative penalties for work safety can increase enterprises’ emphasis on safety. Additionally, the government can not only directly regulate park enterprises to improve work safety conditions but also urge park management to improve the park’s work safety management system and strictly enforce it to promote work safety in park enterprises.

Regression analysis of the factors related to park management influencing safety investment decisions in the park found that the safety management by park management has a positive impact on safety investment in park enterprises.

Therefore, at the park management level, park management needs to implement reasonable safety management for enterprises within the park. Based on the experimental results, park enterprises are willing to increase safety investment to cooperate with park management in work safety. Park management should develop a sound plan for park safety management, guide enterprises in work safety awareness, and promptly grasp the work safety status of park enterprises to promote the sustainable development of safety within the park.

### 5.2 Research contributions

This study has made contributions to the literature on investment decision-making in work safety for industrial parks. Firstly, existing literature has already confirmed that factors such as government work safety supervision, safety costs and safety benefits, and the capability of work safety resources influence the investment decision-making of enterprises in work safety. However, these studies have not considered the characteristics of industrial park enterprises, such as their small economic scale, centralized decision-making authority, and negative externalities of accidents. Therefore, it is still unclear whether these factors affect the investment decision-making of work safety in industrial park enterprises.

This study aims to investigate the factors that influence safety investment decisions in park enterprises and make contributions to existing literature from three perspectives. Firstly, through the use of behavioral experiments, this study allows participants to perceive that investing in safety costs can result in safety benefits, thereby highlighting the tendency of decision-makers to make proactive safety investment decisions when they recognize a positive relationship between work safety investment and safety benefits. Secondly, based on research conducted on park enterprises, this study verifies that decision-makers in park enterprises, with altruistic preferences, increase safety investment in response to the negative external effects of safety accidents within the park. Lastly, while previous studies have primarily focused on developing models to enhance safety investment and reduce accidents, this study employs behavioral experiments to create realistic scenarios. Given that the decision-making authority predominantly lies with enterprise owners in park enterprises, this study specifically targets decision-makers within these enterprises, examining the factors that influence safety investment decisions based on their inherent preferences and the choices they make in response to the realistic scenarios presented in the experiment.

This study primarily focuses on analyzing the characteristics of work safety accidents in industrial park enterprises and their external environment. The main conclusion drawn from this analysis is that improving the work safety situation in industrial parks can be achieved through implementing measures by the government and park management. These measures aim to encourage decision-makers in park enterprises to increase investment in work safety, reduce safety accidents, and mitigate the losses incurred from negative externalities associated with safety accidents.

### 5.3 Research limitations and prospects

This study sets parameters and simulates scenarios to experimentally construct a realistic gap between the investment in work safety by industrial park enterprises and its practical application. Additionally, due to limitations in manpower and financial resources, the number of subjects in the experiment is relatively small. Therefore, in the future, it would be beneficial to select more decision-makers from enterprises to conduct experiments and further investigate the sample data in depth.

## Data availability statement

The original contributions presented in the study are included in the article/supplementary material, further inquiries can be directed to the corresponding author.

## Author contributions

SL: Writing – review & editing. SB: Writing – original draft, Writing – review & editing. DY: Writing – review & editing. JZ: Writing – review & editing.
